# Water stress protection by the arbuscular mycorrhizal fungus *Rhizoglomus irregulare* involves physiological and hormonal responses in an organ‐specific manner

**DOI:** 10.1111/ppl.13854

**Published:** 2023-02-01

**Authors:** David H. Fresno, Helena Solé‐Corbatón, Sergi Munné‐Bosch

**Affiliations:** ^1^ Department of Evolutionary Biology, Ecology and Environmental Sciences, Faculty of Biology University of Barcelona Barcelona Spain; ^2^ Institute of Nutrition and Food Safety (INSA), Faculty of Biology University of Barcelona Barcelona Spain

## Abstract

Arbuscular mycorrhizal fungi may alleviate water stress in plants. Although several protection mechanisms have already been described, little information is available on how these fungi influence the hormonal response to water stress at an organ‐specific level. In this study, we evaluated the physiological and hormonal responses to water stress in above and below‐ground tissues of the legume grass *Trifolium repens* colonized by the arbuscular mycorrhizal fungus *Rhizoglomus irregulare*. Plants were subjected to progressive water stress and recovery. Different leaf and root physiological parameters, as well as phytohormone levels, were quantified. Water‐stressed mycorrhizal plants showed an improved water status and no photoinhibition compared to uncolonized individuals, while some stress markers like α‐tocopherol and malondialdehyde content, an indicator of the extent of lipid peroxidation, transiently increased in roots, but not in leaves. Water stress protection exerted by mycorrhiza appeared to be related to a differential root‐to‐shoot redox signaling, probably mediated by jasmonates, and mycorrhization enhanced the production of the cytokinin *trans*‐zeatin in both roots and leaves. Overall, our results suggest that mycorrhization affects physiological, redox and hormonal responses to water stress at an organ‐specific level, which may eventually modulate the final protection of the host from water stress.

## 1 INTRODUCTION

Drought is one of the major abiotic factors influencing plant development. Climate change is predicted to intensify the effects of drought in the future, affecting both worldwide crop production and plant biodiversity (Challinor et al., 2014; Thuiller et al., 2005). Thus, a deep understanding of the mechanisms underlying plant adaptation to this stress is essential to overcome future challenges. Plants have developed a wide variety of physiological mechanisms and defenses to cope with water deficit, like remodeling their root system to increase water uptake, accumulating compatible osmolytes to reduce water loss, or enhancing antioxidant and hormonal responses (Gupta et al., 2020). Despite these successful adaptation mechanisms, drought eventually has detrimental effects on plant fitness and performance. For instance, while stomata closure effectively prevents water loss through transpiration, it also limits CO_2_ diffusion, reducing photosynthetic activity. Additionally, water stress causes a deficient uptake of soil nutrients, worsening the plant's performance (He & Dijkstra, 2014; Tombesi et al., 2015). As a consequence, additional and complementary mechanisms to cope with drought have evolved in plants to improve nutrient homeostasis during water stress.

A successful strategy to face water stress is the establishment of interactions with beneficial microorganisms, among which arbuscular mycorrhizal fungi (AMF) represent one of the most widespread examples. In this symbiosis, the exploratory capacity of the mycorrhizal extraradical hyphae (ERH) increases water and mineral nutrient uptake, which are later exchanged by plant photoassimilates inside the root through specific fungal structures called arbuscules (Parniske, 2008). Water stress alleviation by AMF has been extensively described and several physiological mechanisms underlying this protection have been unraveled. For instance, plant water status is improved, the activity of (non)‐enzymatic antioxidants increases and the accumulation of compatible osmolytes is altered in comparison to non‐mycorrhizal plants (Abdalla & Ahmed, 2021; Bárzana et al., 2015; Huang et al., 2017; Rehman et al., 2022). Although it is well known that mycorrhization broadly improves plant fitness and performance during water stress, mechanisms underlying AMF‐induced drought tolerance are still relatively unknown, in particular in relation to the redox and hormonal events occurring in different organs and to what extent root‐to‐shoot communication may play a role.

One of the major effects of water stress in plants is the generation of reactive oxygen species (ROS). In the case of green plant tissues, singlet oxygen (^1^O_2_) may damage the D1 protein of photosystem II (PSII) and initiate a cascade of lipid peroxidation events in photosynthetic membranes, eventually leading to damages of the photosynthetic apparatus and, eventually, causing death. However, ROS can also serve a role in redox signaling if produced transiently (Mittler, 2017). ROS production and lipid peroxidation may also take place in other tissues. Vitamin E and its most biologically active form, α‐tocopherol, are unique antioxidants controlling the lipid peroxide level and, therefore, the lipid peroxidation autocatalytic cascade in both photosynthetic and non‐photosynthetic tissues (Farmer & Mueller, 2013; Muñoz & Munné‐Bosch, 2019; Munné‐Bosch et al., 2022). Mycorrhization has been described to prevent photoinhibition in different plant species facing different stressful situations (Gupta et al., 2021; Quiroga et al., 2017; Sheng et al., 2008). Reduction in malondialdehyde (MDA) production, an indicator of lipid peroxidation, is frequent in mycorrhizal plants (Pandey & Garg, 2017; Ren et al., 2019; Salloum et al., 2019). The latter reported that mycorrhizal *Glicine max* plants showed lower contents of both MDA and α‐tocopherol in roots. However, reports dealing with AMF effects in water‐stressed plants are lacking and more research is needed to fully understand how AMF may influence vitamin E, lipid peroxidation and oxylipin signals in plants. Oxylipins are derived from the oxidation of polyunsaturated fatty acids. When originating from plastids, oxylipins, including jasmonates, are considered retrograde signals emanating from plastids to the nucleus that provide essential information about the environment, biotic interactions and/or organ developmental stage (Muñoz & Munné‐Bosch, 2020).

Besides the impact on water status and antioxidant responses of the host plant, AMF colonization influences the plant hormonal response and balance. Several hormones are implicated in the regulation of the plant–AMF interaction. For instance, root‐exudated strigolactones have a positive influence at the early steps of root penetration and establishment, while abscisic acid (ABA) and auxins enhance arbuscule formation and root colonization (Akiyama et al., 2005; Floss et al., 2013; Foo et al., 2013; Pozo et al., 2015). Jasmonates, whose most representative compounds are free jasmonic acid (JA), its precursor 12‐*oxo*‐phytodienoic acid (OPDA) and its biologically active conjugated form, jasmonoyl‐isoleucine (JA‐Ile), have also been reported as regulators of plant–AMF interactions. In fact, mycorrhizal roots normally display higher JA levels, and JA‐insensitive and deficient plants consistently show lower mycorrhization rates (Hause et al., 2002; Isayenkov et al., 2005; Tejeda‐Sartorius et al., 2008). In addition, root colonization and further spread in the root have been proven to be regulated by jasmonates (Wasternack & Feussner, 2018; Wasternack & Hause, 2013). Moreover, cytokinins (CKs) are key hormones regulating shoot growth and root branching, and they are essential for the successful establishment of the plant–AMF interaction (Cosme et al., 2016; Fusconi, 2014). Upon an (a)biotic stress, the previous hormonal relation may be altered. Some studies have pointed out that ABA production, which is a key feature in stomatal closure regulation, is milder in mycorrhizal plants upon water stress, and CKs tend to remain higher in mycorrhizal plants (Adolfsson et al., 2017; Ouledali et al., 2019; Ren et al., 2019). Jasmonates are mainly involved in plant tolerance to water stress and AMF‐mediated induced systemic resistance (ISR), while little is known about how mycorrhization may influence this hormonal group upon water stress (Riemann et al., 2015; Wasternack & Hause, 2013). Further research is then needed to better comprehend how mycorrhization may affect the regulation of those hormones involved in plant development and response to abiotic stress.

In this work, we evaluated the effect of the AMF *Rhizoglomus irregulare* on the performance of the highly mycotrophic and water stress‐sensitive forage legume, *Trifolium repens*, under progressive water stress and during water recovery in leaves and roots. Our objective was to determine whether inoculation with this mycorrhizal fungus successfully protected the plant from water stress, and to unravel the physiological and biochemical mechanisms underlying this protection. For that purpose, we analyzed different physiological parameters and stress marker molecules, like MDA and α‐tocopherol, in leaves and roots. In addition, some fungi have been reported to produce and accumulate vitamin E within their tissues (Barros et al., 2008). To test whether *R. irregulare* synthesizes this molecule, which could potentially influence the root antioxidant status, α‐tocopherol content in ERH was measured. Finally, we quantified the levels of ABA, jasmonates and *trans‐*zeatin (*t*‐Z), a biologically active form of cytokinin, to figure out how mycorrhization may protect the plant through the modulation of these key hormones involved in responses to abiotic stress and plant growth regulation. Special emphasis was given to investigating a putative differential response in roots and shoots.

## 2 MATERIALS AND METHODS

### 2.1 Plant and fungal material

The experiment was carried out under controlled conditions in climatic chambers (Ibercex S.L.; lamps: Philips Master TL‐D 36W/840) at 22°C, with a 16:8 h (day: night) photoperiod and a photosynthetic active radiation (PAR) of 120 μmol m^−2^ s^−1^ at the University of Barcelona during 2021. *Trifolium repens* L. seeds were surface‐sterilized and sown on top of 1 L pots containing a sterile mix of sand:vermiculite (70:30). Mycorrhizal pots contained 6 g of an inoculum of the arbuscular mycorrhizal fungus *Rhizoglomus irregulare* (Blaszk, Wubet, Renker and Buscot) Sieverd, Silva and Oehl comb. nov., isolated from a citrus nursery (Estaun et al., 1994). This inoculum was provided by the Institute of Agrifood Research and Technology (Barcelona, Spain) as a bulk inoculum from pots containing mycorrhizal leek (*Allium porrum*). Non‐mycorrhizal control pots contained the same amount of autoclaved inoculum. To homogenize microbial populations, control pots were supplemented with a microbial 20 μm filtrate of the original AMF inoculum. To induce proper root colonization, seeds were initially irrigated with distilled water for 20 days and, onwards, with a ½ Hoagland solution (Hoagland & Broyer, 1936) containing 10% of the standard phosphorous concentration (0.1 mM). Every pot contained 15 white clover plants.

### 2.2 Irrigation treatments and sampling

Plants were submitted either to well‐watered or progressive water stress conditions. Progressive water stress consisted of a 50% irrigation reduction in comparison to well‐watered plants during the first 7 days (from 0 to 7 days after water stress (DAD)), a 75% reduction from 7 to 14 DAD, and 85% reduction from 14 to 20 DAD, the point of maximum water stress. Afterwards, plants were fully watered again during an irrigation recovery period of 10 days (from 20 to 30 DAD). Six pots (*n* = 6) per experimental condition were used.

Samplings and measurements were performed on fully developed nonsenescent leaves at 0, 7, 14, 20, 24, and 30 DAD repeatedly on the same plants (*n* = 6). To determine the maximal photochemical efficiency of photosystem II (*F*_*v*_/*F*_*m*_), measurements at three different leaves per pot were done with a Mini PAM II yield analyzer at each sampling point. One whole leaf per pot was taken to assess relative water content (RWC) following the formula $\mathrm{RWC}=100\times \left(\mathrm{FW}-\mathrm{DW}\right)/\left(\mathrm{TW}-\mathrm{DW}\right)$, where FW is the fresh weight of the leaf at the sampling date, TW is the turgid mass after rehydration for 24 h in the dark at 4°C, and DW is the dry mass after drying the leaves in an oven until constant weight. Finally, a pool of three whole leaves per pot was taken, immediately frozen in liquid nitrogen and kept at −80°C for further analyses.

Due to plant material limitations, root samplings were only carried out at the beginning of the experiment (0 DAD), at maximum water stress (20 DAD) and at the end of the recovery period (30 DAD). Independent pots to the ones used for leaf measurements were used at each sampling point (*n* = 6). All the roots from each pot were gently cleaned and divided into three subsets. One subset of roots was used to determine root water content (Root WC) using the following formula: $\text{Root}\;\mathrm{WC}=100\times \left(\mathrm{FW}-\mathrm{DW}\right)/\mathrm{FW}$. The roots from the second subset were stained following the ink and vinegar technique described by Vierheilig et al. (1998) and root colonization percentage was calculated according to the gridline‐intersect method (Giovannetti & Mosse, 1980). The remaining subset was immediately frozen in liquid nitrogen, stored at −80°C and freeze‐dried right before performing the biochemical analyses. In addition, fresh and homogeneous soil samples from three pots per experimental conditions (*n* = 3) were weighed and dried in an oven at 70°C until constant weight to finally calculate soil water content (Soil WC) using the following formula: $\text{Soil}\;\mathrm{WC}=100\times \left(\text{SoilFW}-\text{SoilDW}\right)/\text{SoilFW}$.

### 2.3 Hyphal separation and harvesting

An additional independent experiment was performed to determine whether α‐tocopherol, or any other form of vitamin E, was present in *R. irregulare* extraradical hyphae. *T. repens* seeds were sown on top of 1 L pots containing 6 g of *R. irregulare* inoculum and watered with distilled water. After 24 days, root colonization was checked and confirmed. Twenty of those plants were transplanted around a mycorrhizal trap compartment in a new pot. In total, nine mycorrhizal pots were prepared. The mycorrhizal trap compartment was created according to Emmet et al. (2021), with slight modifications. In brief, this compartment consisted of a cylinder made with a 45 μm pore‐sized nylon mesh that allowed only ERH to get in, but not roots. Plants were cultivated for 90 days in a climatic chamber (Ibercex S.L.; lamps: Philips Master TL‐D 36W/840) under a 12:12 h (day:night) photoperiod, at 22°C, a PAR of 120 μmol m^−2^ s^−1^, and irrigated with a ½ Hoagland solution containing 10% of the standard phosphorous concentration (0.1 mM). Finally, ERH from each trap compartment were collected and cleaned using distilled water and tweezers under a dissecting microscope. Finally, three pools (*n* = 3) of 30 mg of ERH from different mycorrhizal compartments were washed with a sterile phosphate‐buffered saline (PBS) solution and kept at 4°C for immediate vitamin E extraction. An example of the mycorrhizal trap compartment and experiment setup can be found in Figure S1 for clarification.

### 2.4 Quantification of malondialdehyde in leaves and roots

To estimate the degree of lipid peroxidation, MDA was quantified in leaves and roots following the protocol described by Hodges et al. (1999). In brief, leaf (50 mg) and freeze‐dried root (30 mg) samples were ground and extracted three times with 80% ethanol containing 0.01% butylated hydroxytoluene (BHT). The extract was incubated at 95°C for 25 min either with a solution of trichloroacetic acid (TCA) and BHT (TBA^−^ solution), or a TCA solution containing BHT and thiobarbituric acid (TBA) (TBA^+^ solution). Afterwards, absorbance at 440, 532, and 600 nm was measured using a microplate spectrophotometer (xMark Bio‐Rad).

### 2.5 Leaf, root and hyphal vitamin E quantification

Ground leaf frozen samples (50 mg) and freeze‐dried roots (30 mg) were extracted with 250 μl of pure methanol, subjected to ultrasonication and vortexing for 30 min, and centrifuged for 10 min at 15,000*g* (PrismR, Labnet International Inc.). The supernatant was collected, and the pellet was extracted two more times using the same procedure. The three supernatants were merged and filtered through a 0.22 μm filter (Phenomenex). In the case of ERH samples, 30 mg of fresh hyphae were transferred to an Eppendorf tube containing 190 mg of round‐shaped 90–150 μm diameter glass beads and 400 μl of pure methanol. ERH were ground by agitation during 4 min at 30 beats/second. The homogenate was subjected to vortexing and ultrasonication for 20 min, followed by maceration for 1 h at 4°C and centrifuged at 13,000 rpm for 10 min. Supernatant was collected. The pellet was extracted again in 100 μl to avoid excessive compound dilution. Both supernatants were merged and filtered through a 0.22 μm filter. Vitamin E compounds (tocopherols and tocotrienols) were separated by HPLC using an Intersil SIL‐100A chromatography column (GL Sciences) and quantified with a fluorescence detector (JASCO FP‐1520) as described by Amaral et al. (2005). A calibration curve was made with standards of the tocopherols and tocotrienols analyzed.

### 2.6 Leaf and root hormone analyses

Plant hormones, including different jasmonates (OPDA, JA and the conjugated form JA‐Ile), ABA and the cytokinin *t*‐Z were extracted and quantified by ultrahigh‐performance liquid chromatography coupled to tandem mass spectrometry (UHPLC–MS/MS). Ground leaves (50 mg) and freeze‐dried roots (30 mg) were extracted with 250 μl of a methanol:2‐propanol:glacial acetic acid (50:49:1) mix and deuterium‐labeled standards (d5‐JA, d6‐ABA, and d5‐tZ). The extracts were subjected to ultrasonication (Branson 2510 ultrasonic cleaner, Bransonic) and vortexing for 30 min, followed by a 10 min centrifugation at 15,000 *g* (PrismR, Labnet International Inc.). The supernatant was collected, and the pellet was reextracted. Both supernatants were merged and filtered through a hydrophobic 0.22 μm filter (Phenomenex). Hormone levels were analyzed by UHPLC‐ESI‐MS/MS as described by Müller and Munné‐Bosch (2011). The UHPLC was coupled to a triple quadrupole mass spectrometer (QTRAP 4000, AB Sciex). A LUNA C18 column (Phenomenex, 1.6 μm, 100 × 2.1 mm) was used. Solvent A was water with 0.05% acetic acid and solvent B was acetonitrile with 0.05% acetic acid. Flow rate was set at 0.6 ml min^−1^. Quantification was made considering recovery rates for each sample by using the deuterium‐labeled internal standards. Calibration curves for each analyte were generated using MultiQuantTM 3.0.1 software.

### 2.7 Statistical analyses

The effects of the factors “Time,” “Mycorrhization,” and “Water Regime” and their interactions were analyzed. For leaf data, as measurements were taken in a repetitive fashion on the same pots, a linear mixed effect model for repeated measures was used, considering “Plant Individual” as a random effect. For root analyses, as independent pots were used at every sampling point, a three‐way ANOVA with interaction was performed, followed by a post hoc Duncan test. Data were transformed for statistical analyses whenever necessary to achieve normal distribution and homoscedasticity of residuals; except for root colonization percentage, where any transformation worked. Consequently, individual nonparametric Kruskal–Wallis tests between experimental conditions were performed. In all cases, differences were considered significant at a probability level of *p* < 0.05. Summarized statistical information is depicted in every figure.

As different parameters were measured on leaves and roots, specific Spearman rank correlation tests were performed for each tissue using a total of 144 observations for leaves (*n* = 144) and 72 for roots (*n* = 72). For those parameters that were shared both by leaves and roots (i.e., MDA, α‐tocopherol and hormone levels), a whole Spearman correlation was performed, merging both root and leaf data, with a total of 216 observations (*n* = 216). Finally, correlation analyses between root and leaf parameters were performed. All analyses were performed using RStudio (RStudio Team, 2020).

## 3 RESULTS

### 3.1 Mycorrhization by *Rhizoglomus irregulare* protects *Trifolium repens* from progressive water stress

**FIGURE 1:**
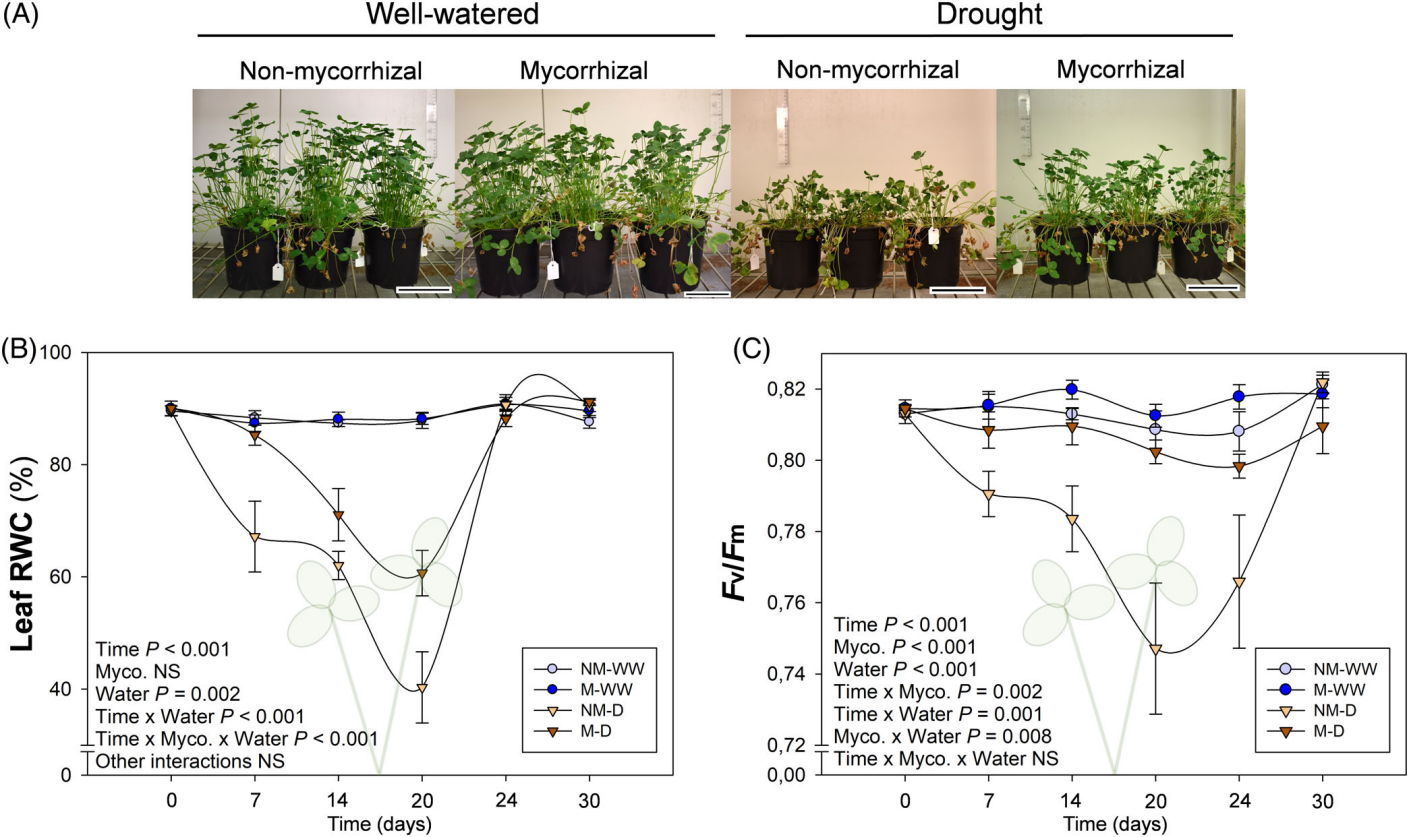
Mycorrhization protects *Trifolium repens* from progressive water stress. Mycorrhizal (M) and non‐mycorrhizal (NM) plants were well‐watered (WW) or submitted to progressive water stress (D) up to 20 days, followed by irrigation recovery for 6 days. (A) Representative plant samples of each treatment at maximum water stress point, 20 days after the beginning of the treatment. (B) Leaf relative water content (RWC) and (C) maximum photosynthetic efficiency of photosystem II (*F*_*v*_/*F*_*m*_) are represented. Data are means ± SEM of *n* = 6 pots, each being a pool of 15 plants. To evaluate treatment effects, a linear mixed effect model for repeated measures was performed. Scale bar corresponds to 10 cm

Progressive water stress was shown to significantly affect plant fitness of both mycorrhizal and non‐mycorrhizal white clover plants, and mycorrhization substantially alleviated this stress. Although water‐stressed mycorrhizal plants showed a weakened phenotype in comparison to unstressed plants, they were in better condition than non‐mycorrhizal individuals (Figure 1A). Something similar occurred with leaf RWC. Non‐mycorrhizal plants displayed a quick drop in leaf RWC starting as soon as 7 DAD and going down to levels of 40% at the point of maximum stress (20 DAD). On the contrary, this drop was delayed in mycorrhizal plants, starting at 14 DAD, and less intense than in non‐mycorrhizal plants (Figure 1B). In both cases, RWC quickly bounced back to normal levels during the irrigation recovery period. The *F*_*v*_/*F*_*m*_ ratio dropped in non‐mycorrhizal stressed plants, reaching values around 0.74–0.75, close to photoinhibition, at 20 DAD. Its levels fully recovered when irrigation was resumed. On the contrary, no variations were observed in water‐stressed mycorrhizal plants, which had similar levels to those of unstressed plants throughout the whole experiment (Figure 1C).

**FIGURE 2:**
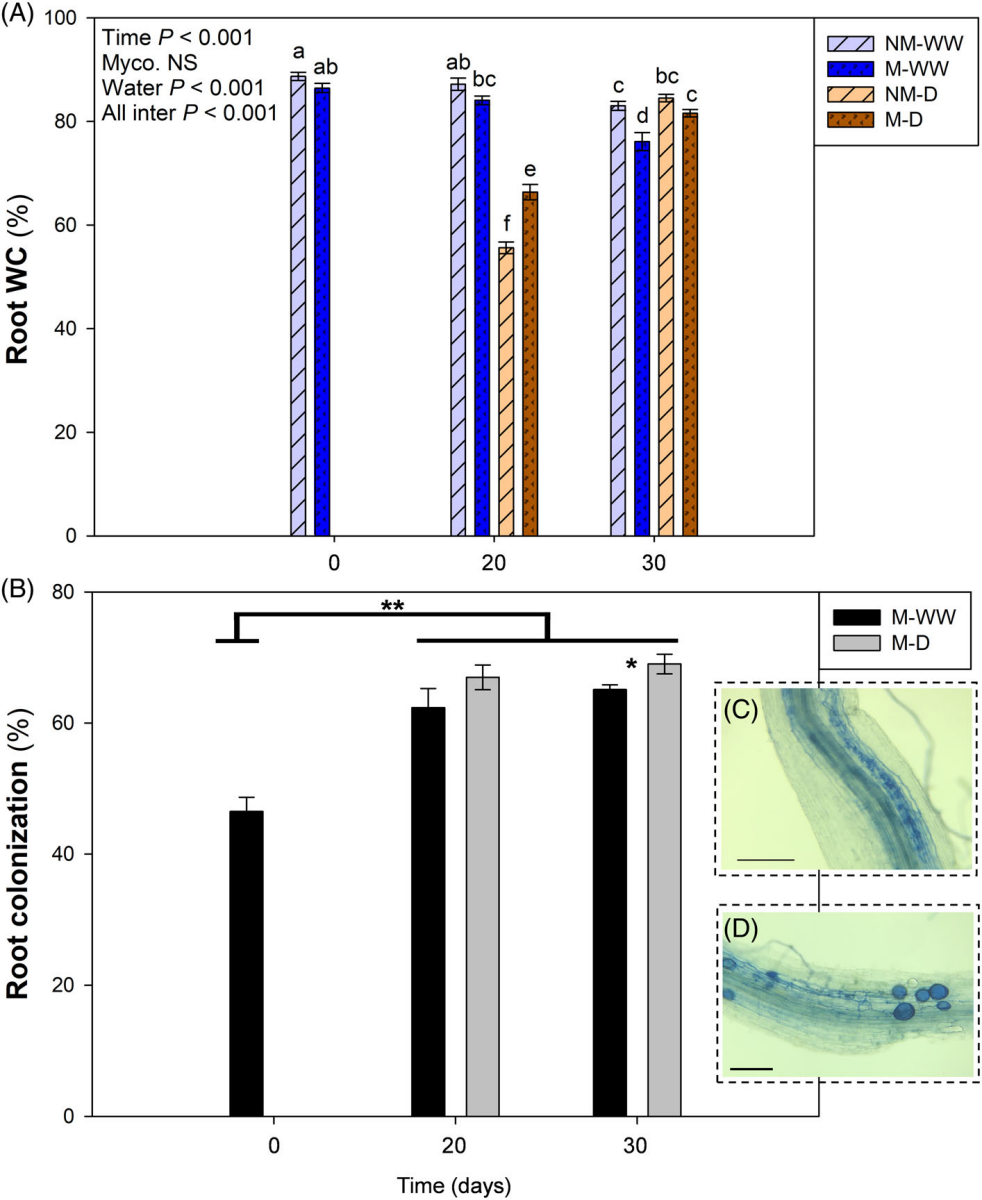
Colonization by *Rhizoglomus irregulare* improves the root water status. Mycorrhizal (M) and non‐mycorrhizal (NM) *Trifolium repens* plants were well‐watered (WW) or submitted to progressive water stress (D) up to 20 days, followed by a water recovery period of 6 days. (A) Root Water Content (WC) and (B) root colonization percentage (%) are represented at the beginning of the water stress period (0 days), maximum water stress (20 days) and end of recovery period (30 days). Mycorrhizal structures like arbuscules and vesicles are shown in panels C and D, respectively. Data are means ± SEM of *n* = 6 pots, each being a pool of 15 plants. For Root WC, treatment effects were evaluated using a three‐way ANOVA for independent samples, followed by a post hoc Duncan test. Different letters indicate significant differences between groups (*p* < 0.05). For root colonization, Kruskal–Wallis nonparametric tests were performed between groups, and significant differences are represented with asterisks (*p < 0.05; **p < 0.01). Scale bars correspond to 200 μm

Protection by mycorrhization was extended to roots. Root water content of stressed plants was significantly lower in comparison to well‐watered plants at 20 DAD, although it was slightly higher in mycorrhizal than in non‐mycorrhizal plants, and in both cases it returned to standard levels after the irrigation recovery period (Figure 2A). Root colonization by *R. irregulare* was abundant, and it increased throughout the experiment regardless of the water treatment, especially from 0 to 20 DAD (Figure 2B). Diverse mycorrhizal structures of a functional mycorrhizal symbiosis, like arbuscules and vesicles, were recurrently observed under a dissecting microscope (Figure 2C,D). Non‐mycorrhizal plants showed no fungal structures in the roots.

### 3.2 Lipid peroxidation in roots increases upon colonization by *Rhizoglomus irregulare*

**FIGURE 3:**
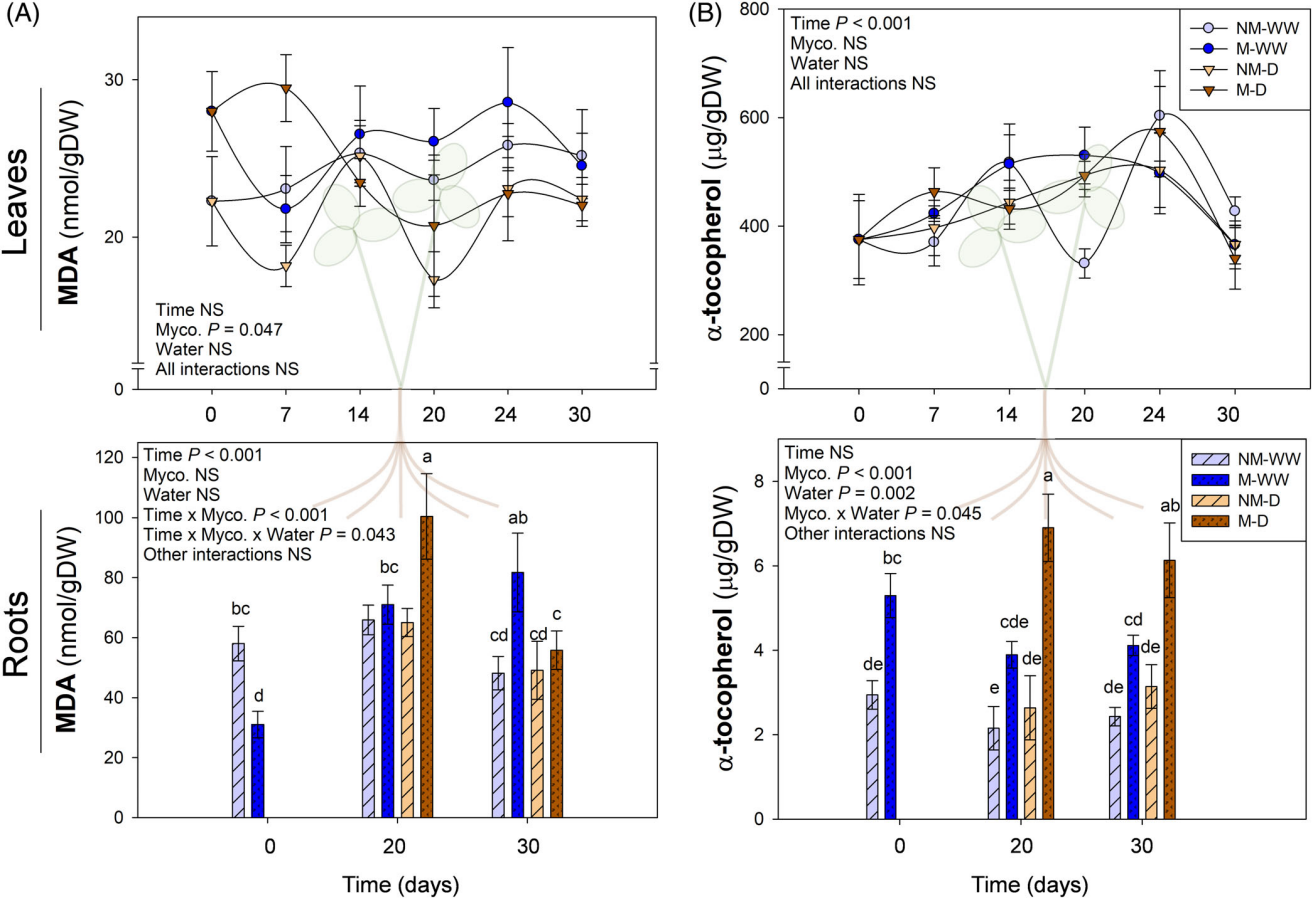
Variation of malondialdehyde (MDA) (A) and α‐tocopherol (B) contents in leaves and roots of mycorrhizal (M) and non‐mycorrhizal (NM) *Trifolium repens* plants under well‐watered (WW) and water stress (D) conditions. Top and bottom panels correspond to leaf and root hormone levels, respectively. Samplings were performed during progressive water stress up to 20 days after the beginning of the treatment, followed by a recovery period of 6 days. Data are means ± SEM of *n* = 6 pots, each being a pool of 15 plants. To evaluate treatment effects in leaf analyses, a linear mixed effect model for repeated measures was applied, while a three‐way ANOVA for independent samples was performed for root data. In the case of root data analyses, a post hoc Duncan test was performed, with different letters indicating significant differences between groups (*p* < 0.05).

MDA concentration was evaluated in leaves and roots as an indicator of the extent of lipid peroxidation. Leaf MDA remained quite stable throughout time, and no relevant effect of any factor was observed (Figure 3A). Similarly, α‐tocopherol followed a comparable pattern to that of MDA. Only “Time” had a significant effect on its concentration, but neither mycorrhization nor water stress affected leaf tocopherol content. On the contrary, root MDA levels were influenced by mycorrhization. While non‐mycorrhizal plants displayed relatively stable levels of MDA over time, mycorrhization seemed to consistently increase MDA levels throughout the experiment. Noteworthy, water‐stressed mycorrhizal plants showed a 3‐fold increase at 20 DAD that rapidly came back to standard levels during the water recovery period. In well‐watered mycorrhizal roots, however, although MDA rise was lower than in water‐stressed individuals (2‐fold increase), their levels remained high also during the water recovery period. In contrast, root MDA levels recovered fully after 10 days of recovery (30 DAD); thus, showing that the 3‐fold increase observed in root MDA levels in water‐stressed mycorrhizal plants was fully transient (Figure 3A). Regarding α‐tocopherol, it was more abundant in mycorrhizal roots prior to water stress (0 DAD), and this effect was reinforced in stressed mycorrhizal plants at the point of maximum stress (20 DAD), with a 2‐fold increase in comparison to non‐mycorrhizal stressed roots. This trend was maintained throughout the recovery period (30 DAD) (Figure 3B). To investigate whether the α‐tocopherol increase in mycorrhizal roots occurred due to the presence of this antioxidant in the fungus itself, further biochemical analysis of *R. irregulare* extraradical mycelium was performed. No α‐tocopherol was detected, confirming that it was being synthesized by the host.

### 3.3 Mycorrhization affects the jasmonate response in front of water stress

**FIGURE 4:**
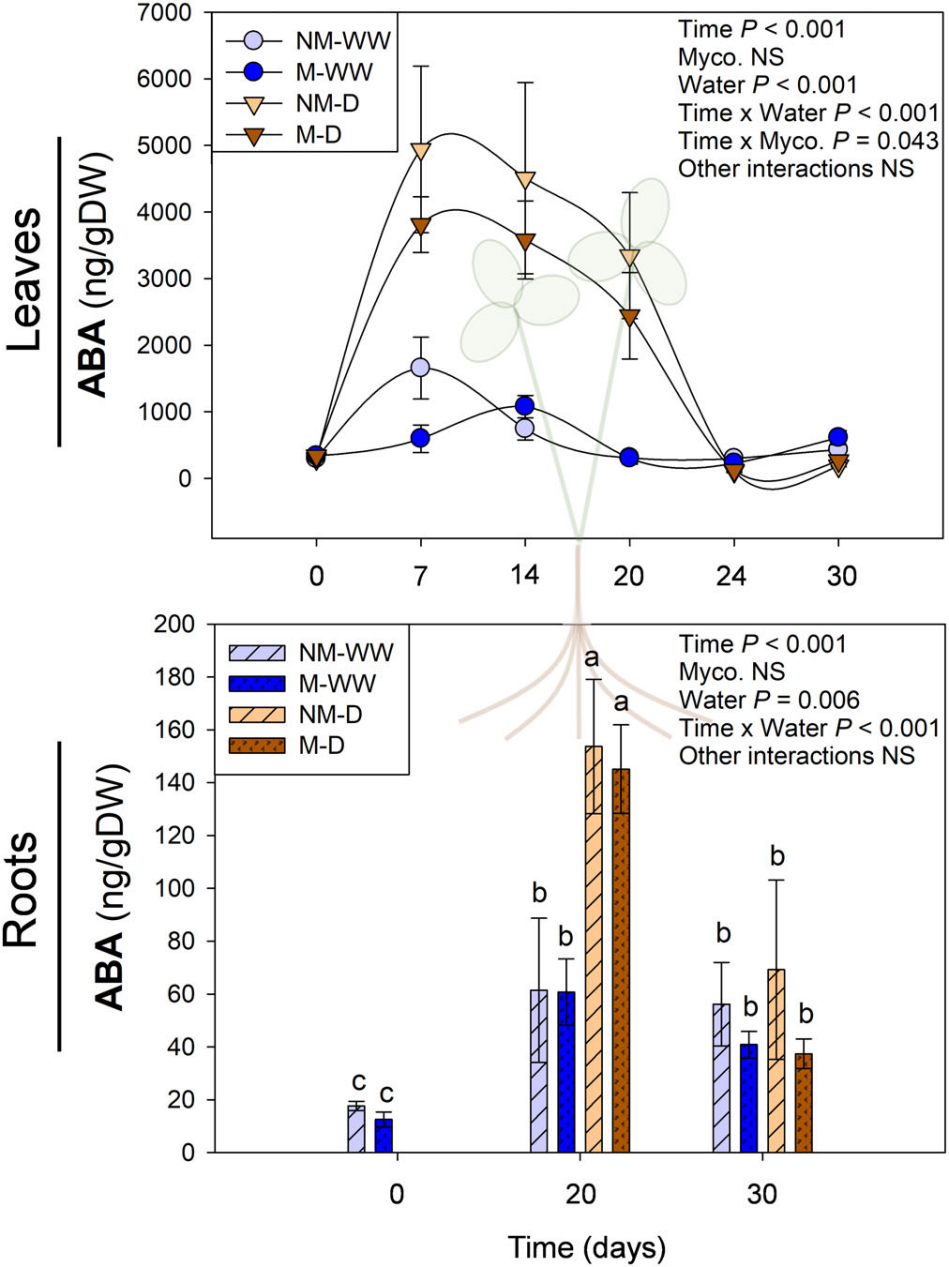
Content of abscisic acid (ABA) in leaves and roots of mycorrhizal (M) and non‐mycorrhizal (NM) *Trifolium repens* plants under well‐watered (WW) and water stress (D) conditions. Top and bottom panels correspond to leaf and root hormone levels, respectively. Samplings were performed during progressive water stress up to 20 days after the beginning of the treatment, which was followed by recovery for 6 days. Data are means ± SEM of *n* = 6 pots, each being a pool of 15 plants. To evaluate treatment effects, a linear mixed effect model for repeated measures was performed for leaf hormone contents, while a three‐way ANOVA for independent samples was applied for root hormone data, followed by a post hoc Duncan test. Different letters indicate significant differences between groups (*p* < 0.05)

Mycorrhization influenced cytokinin levels regardless of water stress, the latter enhanced ABA irrespective of mycorrhization, and finally, mycorrhization affected the jasmonate response upon water stress. Leaf ABA concentration steeply increased at 7 DAD and remained at high levels throughout the water stress, falling back to standard levels during water recovery (Figure 4). No significant effect of “Mycorrhization” was observed, and only its interaction with “Time” was slightly significant. Root response was water dependent as in leaves, with increased levels of ABA at maximum water stress in comparison to well‐watered plants. ABA concentration came back to normal levels after irrigation recovery. No significant effect of “Mycorrhization,” or any of its interactions with other factors, was significant. Therefore, water stress enhanced ABA production both in leaves and roots, and mycorrhization by *R. irregulare* did not significantly influence this response (Figure 4).

**FIGURE 5:**
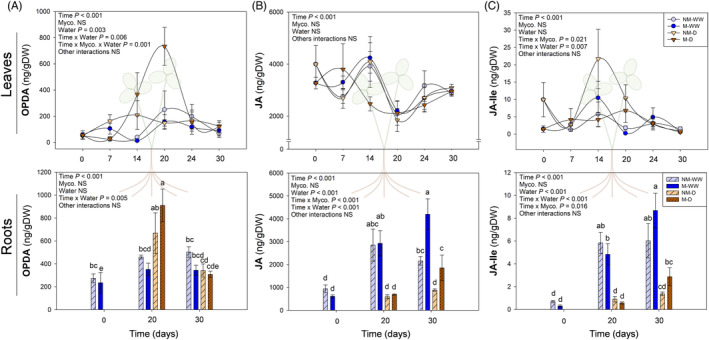
Content of jasmonates, including 12‐*oxo*‐phytodienoic acid (OPDA) (A), jasmonic acid (JA) (B) and jasmonoyl‐isoleucine (JA‐Ile) (C) in leaves and roots of mycorrhizal (M) and non‐mycorrhizal (NM) *Trifolium repens* plants under well‐watered (WW) and water stress (D) conditions. Top and bottom panels correspond to leaf and root hormone levels, respectively. Samplings were performed during progressive water stress up to 20 days after the beginning of the treatment, which was followed by recovery for 6 days. Data are means ± SEM of *n* = 6 pots, each being a pool of 15 plants. To evaluate treatment effects, a linear mixed effect model for repeated measures was performed for leaf hormone contents, while a three‐way ANOVA for independent samples was applied for root hormone data, followed by a post hoc Duncan test. Different letters indicate significant differences between groups (*p* < 0.05)

Mycorrhization and water stress significantly altered jasmonate profile both in leaves and roots. In the case of leaves, a clear accumulation of the precursor OPDA was observed in water‐stressed mycorrhizal plants, with three times higher levels than non‐mycorrhizal stressed plants, at maximum water stress (20 DAD). The statistical analysis revealed a significant effect of “Time,” “Water Regime,” their interaction, and the triple interaction between “Time,” “Mycorrhization,” and “Water Regime” (Figure 5A). Non‐mycorrhizal water‐stressed individuals behaved similarly to unstressed plants, with no significant variations in OPDA throughout the experiment. No significant fluctuations in free JA or the biologically active JA‐Ile were observed in mycorrhizal stressed plants, or in any other experimental group (Figure 5B,C). Roots displayed similar patterns to those observed in leaves, although the effect of water stress on the hormonal response here was more abrupt. Water stress provoked an accumulation of OPDA, especially in mycorrhizal plants (Figure 5A). During recovery, its levels came back to standard concentrations. This increase in OPDA in front of water stress contrasted with much lower levels of JA or JA‐Ile at 20 DAD, either for mycorrhizal or non‐mycorrhizal roots. Only during water recovery, mycorrhizal stressed plants slightly returned to their normal JA levels (Figure 5B).

**FIGURE 6:**
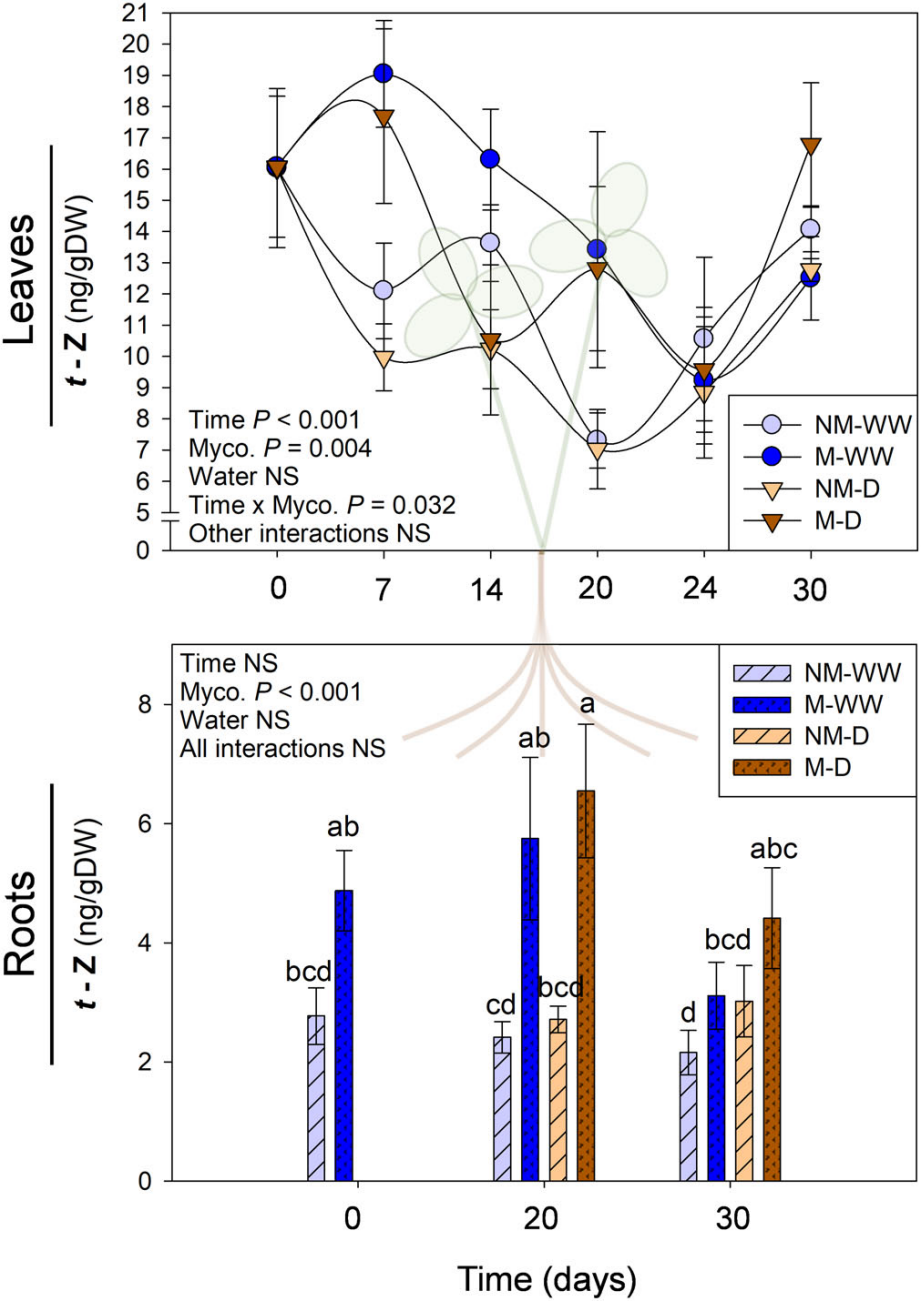
Content of *trans‐*zeatin (*t*‐Z) in leaves and roots of non‐mycorrhizal (NM) and mycorrhizal (M) *Trifolium repens* plants under well‐watered (WW) and water stress (D) conditions. Top and bottom panels correspond to leaf and root hormone levels, respectively. Samplings were performed during progressive water stress up to 20 days after the beginning of the treatment, which was followed by recovery for 6 days. Data are means ± SEM of *n* = 6 pots, each being a pool of 15 plants. To evaluate treatment effects, a linear mixed effect model for repeated measures was performed for leaf hormone contents, while a three‐way ANOVA for independent samples was applied for root hormones data, followed by a post hoc Duncan test. Different letters indicate significant differences between groups (*p* < 0.05)

Mycorrhization promoted the production of the active cytokinin *t*‐Z both in leaves and roots in a water‐independent manner (Figure 6). Leaves of mycorrhizal *T. repens* consistently displayed higher levels of this hormone throughout the experiment, especially at 7 and 20 DAD. In general, this hormone tended to decrease over time, followed by a pullback to higher levels at the end of the experiment. The *t*‐Z levels began to fall as soon as 7 DAD in non‐mycorrhizal plants, while this decrease was delayed by 7 more days in mycorrhizal individuals. In addition, *t*‐Z was higher in mycorrhizal plants at 20 DAD. “Time,” “Mycorrhization” and their interaction effects were significant. No differences were found in leaf *t*‐Z levels between stressed and unstressed mycorrhizal plants, except at 14 DAD, which suggests a water stress‐independent regulation of cytokinin production. A similar response was observed in roots, where mycorrhizal plants, regardless of the water regime applied, showed significantly higher levels of *t*‐Z in comparison to their control. This was especially visible at 0 and 20 DAD, while no differences were observed after the recovery period.

## 4 DISCUSSION

Plants have developed different mechanisms to face abiotic stresses like drought, and their association with beneficial microbes, like arbuscular mycorrhizal fungi, can modify and improve plant performance in front of such stresses (Gupta et al., 2020; Huang et al., 2017). In this experiment, the protective effect of the AMF *R. irregulare* in front of a progressive water stress in *T. repens* was tested. Mycorrhization improved the water status of leaves and roots, and prevented photoinhibition during water stress. As a consequence, mycorrhizal plants showed a better condition than noncolonized individuals when facing water stress (Figures 1 and 2). The protection effect observed in this experiment has been extensively described before in different species, although many of the specific mechanisms underlying it were still unknown (Abdalla & Ahmed, 2021). Here, we provide evidence that lipid peroxidation‐derived signals, that is oxylipins, such as malondialdehyde and jasmonates, transiently increased in roots of water‐stressed plants upon mycorrhization and we suggest that the water stress protection exerted by mycorrhiza may be related to a differential root‐to‐shoot redox signaling, probably mediated by jasmonates.

Water stress usually provokes ROS generation, which may lead to membrane lipid peroxidation and, eventually, tissue damage and malfunctioning of the photosynthetic apparatus (Farmer & Mueller, 2013). In different plant species, AMF generally reduce ROS production and lipid peroxidation through the activation of several enzymatic and nonenzymatic antioxidants (Bahadur et al., 2019; Pandey & Garg, 2017). However, the benefits and damage of this response may be strongly organ‐ and time‐specific. Depending on the organ in which lipid peroxidation occurs and if this occurs transiently or sustained in time, lipid peroxidation can reflect a tolerance mechanism or represent an inflicted damage. In our experiment, leaf MDA was hardly altered by water stress over time, suggesting that this organ was well protected from lipid peroxidation (Figure 3A, top panel). Furthermore, photoinhibition was very mild (with minimum *F*_*v*_/*F*_*m*_ values of 0.75 in water‐stressed plants) and transient, thus indicating white clover was quite tolerant to photoinhibition, despite the significant reductions observed in the RWC. In addition, *R. irregulare* inoculation led to full protection of the photosynthetic apparatus, maintaining photosystem II stability (with values above 0.80) upon water stress (Figure 1C); thus indicating that white clover leaves are tolerant to water stress, most particularly when mycorrhization occurs, as already described in other species (Gupta et al., 2021; Moustakas et al., 2020).

Interestingly, in roots, water stress did not induce an increased lipid peroxidation by itself. However, the combination of water stress and mycorrhization led to a sharp production of MDA that quickly came back to background levels during the water recovery period, in contrast to well‐watered mycorrhizal plants, where MDA remained high during recovery (Figure 3A, bottom panel). This divergence in MDA patterns between stressed and well‐watered mycorrhizal plants could be explained by the differential response observed in α‐tocopherol production, which was significantly higher in the former, even during recovery (Figure 3B, bottom panel). Vitamin E, and especially α‐tocopherol, is the main antioxidant capable of modulating the production of lipid peroxidation‐derived signals and restricting uncontrolled lipid peroxidation (Muñoz & Munné‐Bosch, 2019). Its increase in water‐stressed mycorrhizal plants may explain the decrease in MDA during water recovery. The biochemical analysis of *R. irregulare* ERH discarded the fungal origin of the rise in α‐tocopherol. Altogether, our results show that the combination of mycorrhiza and water stress‐driven lipid peroxidation may activate local α‐tocopherol production in roots, leading to a better antioxidant status of the roots even after the stress is over. This could be considered as an “antioxidant priming” induced by *R. irregulare* in combination with water stress.

Besides antioxidants, phytohormones also play a key role in plant defense to abiotic stresses. For instance, a common response to water stress is a sharp and quick increase in ABA. This hormone is one of the main mediators in plant responses to water stress through the regulation of stomatal closure and root growth (Cutler et al., 2010; Zhang et al., 2022). Previous studies in different species have shown that mycorrhizal plants may have higher or lower ABA production in front of abiotic stresses in comparison to non‐mycorrhizal stressed plants. This points out the potential role of this hormone as a mediator of water stress alleviation by AMF (Aroca et al., 2013; Chitarra et al., 2016; Xie et al., 2018). In our experiment, *T. repens* plants presented a clear increase in ABA content both in leaves and roots during the dry period (Figure 4). However, no significant differences in ABA dynamics were detected between mycorrhizal and non‐mycorrhizal plants, suggesting that AMF‐mediated protection may be independent of ABA contents in white clover in both plant organs. However, further research is needed to identify possible targets in ABA signaling.

In addition to ABA, jasmonates are key regulatory hormones in plant responses to (a)biotic stress, and they are tightly linked to plant–AMF interactions, regulating the fungal colonization of the root. In our experiment, low JA and JA‐Ile concentrations in stressed roots suggest that water stress drastically blocked OPDA transformation into free and biologically active jasmonate forms in *T. repens* roots (Figure 5, bottom panels), and this biochemical transformation from OPDA to JA and then JA‐Ile was barely influenced by mycorrhization. Surprisingly, *R. irregulare* had a significant effect on jasmonate dynamics in leaves. While non‐mycorrhizal water‐stressed plants behaved similarly to well‐watered plants, mycorrhization induced a strong accumulation of OPDA in response to water stress (Figure 5, top panels). This suggests that this hormone may be involved in AMF‐induced tolerance to water stress in white clover. On the other hand, this accumulation may suggest that an OPDA‐mediated defense signaling to water stress could be taking place mainly in mycorrhizal stressed plants. Indeed, water stress induced OPDA accumulation in roots, most particularly in mycorrhizal plants and OPDA contents increased sharply in leaves of water‐stressed mycorrhizal plants only (Figure 5), thus supporting the contention that OPDA might be transported through the xylem from roots to shoots to activate a defensive signaling response. Although we did not study jasmonate contents in the xylem, previous studies have shown that xylem‐borne jasmonates are biologically active; so, xylem OPDA can play a role as a jasmonic acid precursor and putative antitranspirant (De Ollas et al., 2018). Although OPDA can also be transported by the phloem (Koenig & Hoffmann‐Benning, 2020), our results indicate that water stress might be causing first an increase of jasmonates in roots and then mycorrhization facilitates its transport to the shoots, not counterwave, since water stress in non‐mycorrhizal plants did not lead to increased OPDA contents in leaves (Figure 5A). Although JA‐Ile is normally the principal jasmonate involved in responses to (a)biotic stresses through the transcription activation of JA‐responsive elements, OPDA can also act as an alternative signaling stress molecule, triggering specific adaptive responses (e.g., heat shock transcription factors) that enhance plant tolerance to stress (Ali & Baek, 2020; Riemann et al., 2015; Savchenko et al., 2014; Seo et al., 2011; Taki et al., 2005). In addition, OPDA and ABA have been proven to act synergistically in stomatal closure regulation, improving plant water status (Savchenko et al., 2014). Altogether, mycorrhization seems to influence the jasmonate response to water stress especially through the accumulation of OPDA.

Mycorrhization also influences plant growth. Cytokinins are essential regulators of plant growth both in shoots and roots, and they are considered reliable markers of plant vigor. Besides, they are also necessary to establish successful plant–AMF interactions (Cosme et al., 2016; Fusconi, 2014). In this experiment, *R. irregulare* consistently increased *t*‐Z content in roots and leaves throughout the experiment in a water stress‐independent manner (Figure 6). This observation coincides with the one made by Adolfsson et al. (2017), where mycorrhization of the legume *Medicago truncatula* by *Rhizophagus irregularis* caused an increase in different CKs under no stress conditions. Higher *t*‐Z levels in mycorrhizal plants may explain a constitutive growth promotion by the AMF in the case of the shoot, regardless of the abiotic stress conditions. In addition, the higher demand for photoassimilates by fungal arbuscules may increase the “sink strength” of mycorrhizal roots, which may explain higher CK levels (Fusconi, 2014).

**FIGURE 7:**
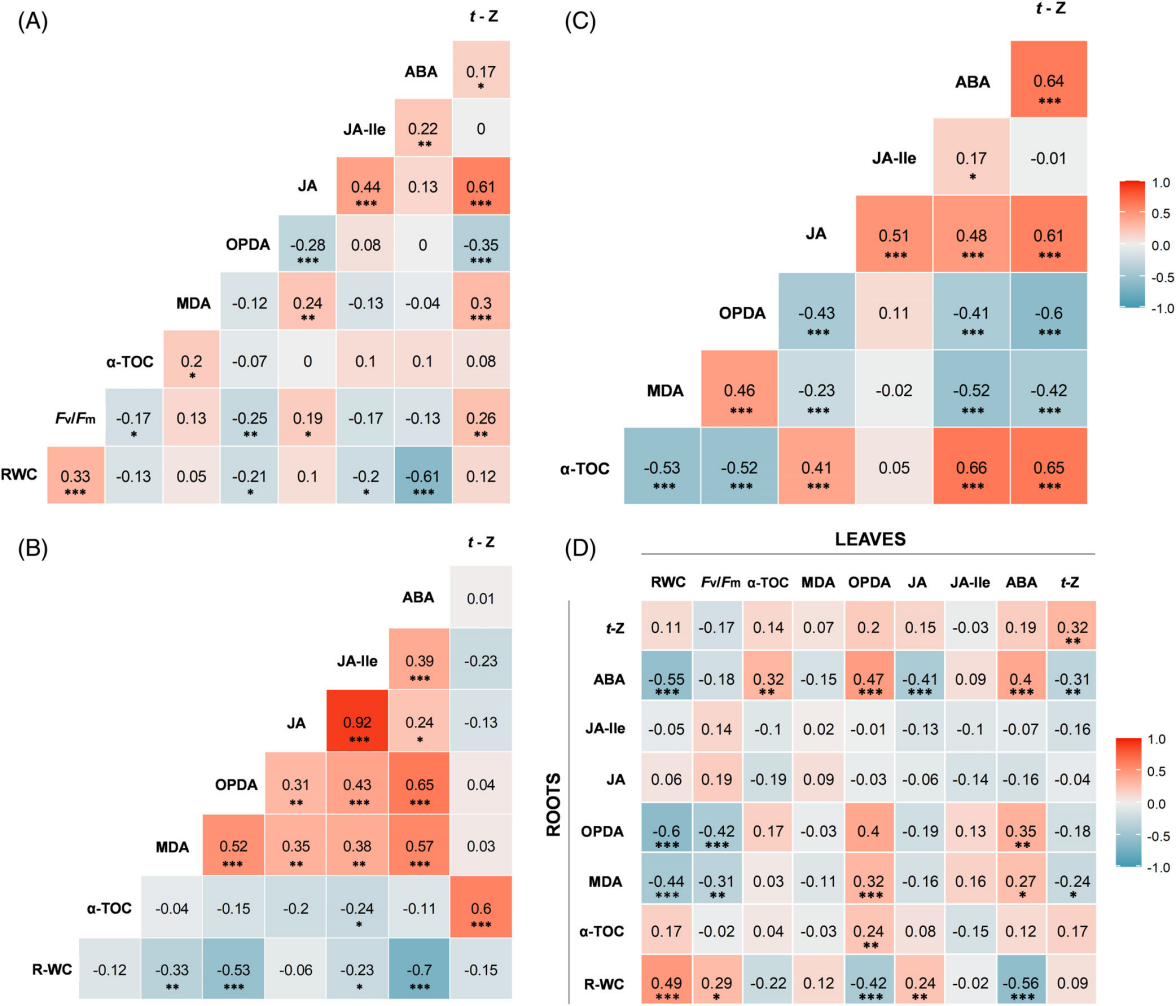
Spearman rank correlation matrices showing rho values between different studied variables. Specific correlation analyses for leaf and root data are represented in graphs (A) and (B), respectively (*n* = 144 measurements for leaves; *n* = 72 measurements for roots). Correlations between malondialdehyde (MDA), α‐tocopherol (α‐TOC) and hormone levels were additionally analyzed, combining both leaf and root data (C) (*n* = 216 measurements). Finally, specific correlations between root and leaf parameters are depicted in graph (D) (*n* = 72 measurements). One, two and three asterisks indicate *p* values below 0.05, 0.01 and 0.001, respectively. The color gradient represents ranges of correlation from red (positive) to blue (negative). ABA, abscisic acid; *F*_*v*_/*F*_*m*_, maximum photochemical efficiency of photosystem II; JA, jasmonic acid; JA‐Ile, jasmonoyl‐isoleucine; OPDA, 12‐*oxo*‐phytodienoic acid; RWC, relative water content; R‐WC, root water content; *t*‐Z, *trans*‐zeatin

Finally, we performed correlation analyses between the variables examined (Figure 7). Considering significant and strong Spearman correlations between the variables examined (considering rho values above 0.60 combined with *p* values below 0.001 only, according to Zuur et al., 2009), we found that the RWC strongly negatively correlated with ABA contents, and that ABA strongly positively correlated with *t*‐Z in leaves (Figure 7A), Also, we found that the root water content negatively correlated with root ABA levels, *t*‐Z positively correlated with α‐tocopherol, ABA positively correlated with OPDA and JA with JA‐Ile in roots (Figure 7B). Furthermore, we found that both ABA and *t*‐Z contents strongly positively correlated with *t*‐Z (while OPDA strongly negatively correlated with *t*‐Z), and that both ABA and JA contents strongly positively correlated with α‐tocopherol by pooling data from both organs (Figure 7C). Finally, we found that root OPDA strongly negatively correlated with leaf RWC (Figure 7D). The latter result is particularly interesting since it is shown that jasmonates in roots might have a positive effect in preserving water in roots, which confirms the aforementioned role of OPDA as an antitranspirant and, most importantly, suggests that this effect in the shoot is triggered by root borne signals. Considering that OPDA is an oxylipin that results from the oxidation of fatty acids from plastids, these results suggest that signals emanating from plastids in roots can modulate responses systemically in leaves, an aspect that warrants further investigation. Furthermore, unfortunately, the effects of mycorrhization on such relationships could not be explored since the *n* values were too low to provide sufficiently strong statistical power.

Overall, our data show that mycorrhization of *T. repens* by the arbuscular mycorrhizal fungus *R. irregulare* effectively ameliorates plant tolerance to progressive water stress, both by improving water status and reducing photoinhibition. The influence of the fungus regarding lipid peroxidation and vitamin E was restricted to the roots, where a clear “antioxidant priming” effect was induced by mycorrhization. On the other hand, mycorrhization may most likely not be acting on ABA but on jasmonates, especially in leaf OPDA (probably originating at least in part from roots), and in *t*‐Z, both in roots and leaves, to improve plant performance. Altogether, plant protection by *R. irregulare* depended on a combined physiological and hormonal regulation with differential responses in leaves and roots.

## AUTHOR CONTRIBUTIONS

David H. Fresno and Sergi Munné‐Bosch conceived and designed the experiments. David H. Fresno and Helena Solé‐Corbatón performed the experiments. David H. Fresno wrote the manuscript with the help of Sergi Munné‐Bosch. All the authors contributed to the discussion and revised and approved the final manuscript.

## CONFLICT OF INTEREST

The authors declare that they have no known competing financial interests or personal relationships that could have appeared to influence the work reported in this paper.

## Supporting information


**Figure S1.** Experimental setup for extraradical hyphae isolation.
**Figure S2.** Soil water content of pots containing mycorrhizal and non‐mycorrhizal *Trifolium repens* plants under well‐watered and water stress conditions.

## Data Availability

The data that support these findings are available from the authors upon reasonable request.
