# Supplementary material for: Water stress protection by the arbuscular mycorrhizal fungus Rhizoglomus irregulare involves physiological and hormonal responses in an organ‐specific manner
Source: Physiol Plant. 2023 Feb 1;175(1):e13854. doi: 10.1111/ppl.13854 (PMC10108154; doi:10.1111/ppl.13854)
Supplement: Supplementary file 1 — Figure S1. Experimental setup for extraradical hyphae isolation. Figure S2. Soil water content of pots containing mycorrhizal and non‐mycorrhizal Trifolium repens plants under well‐watered and water stress conditions. [file PPL-175-0-s001.pdf]

Supplementary Material

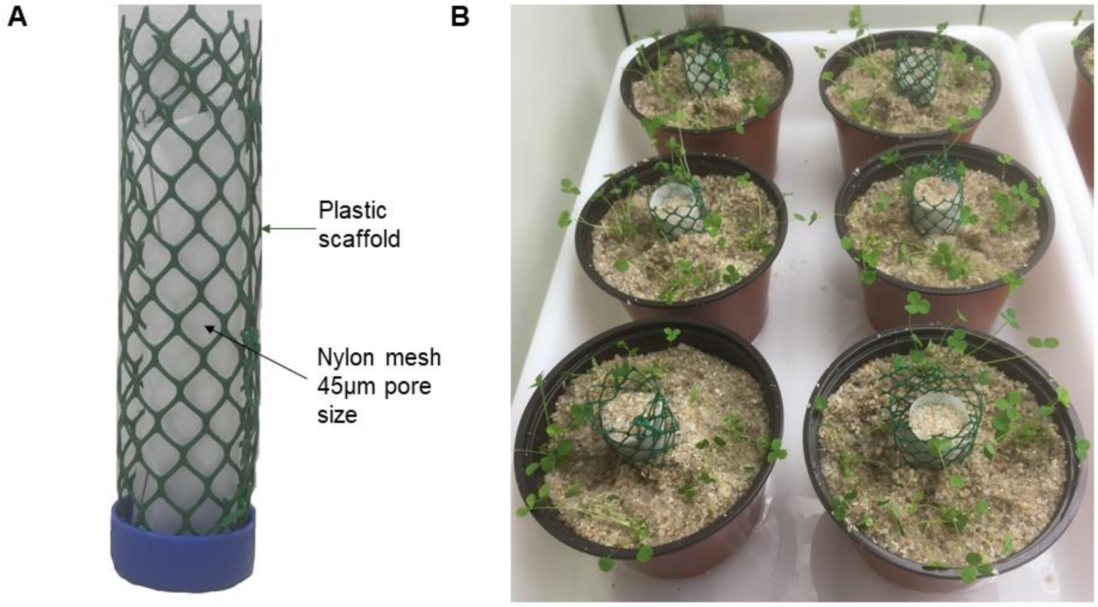

Supp. Figure 1. Experimental setup for extraradical hyphae (ERH) separation. (A) The mycorrhizal trap compartment consisted of a 45µm pore-sized nylon mesh surrounded by a plastic scaffold. The compartment was filled with 2mm autoclaved sand. (B) Mycorrhizal *Trifolium repens* plantlets were planted around the mycorrhizal trap compartment.

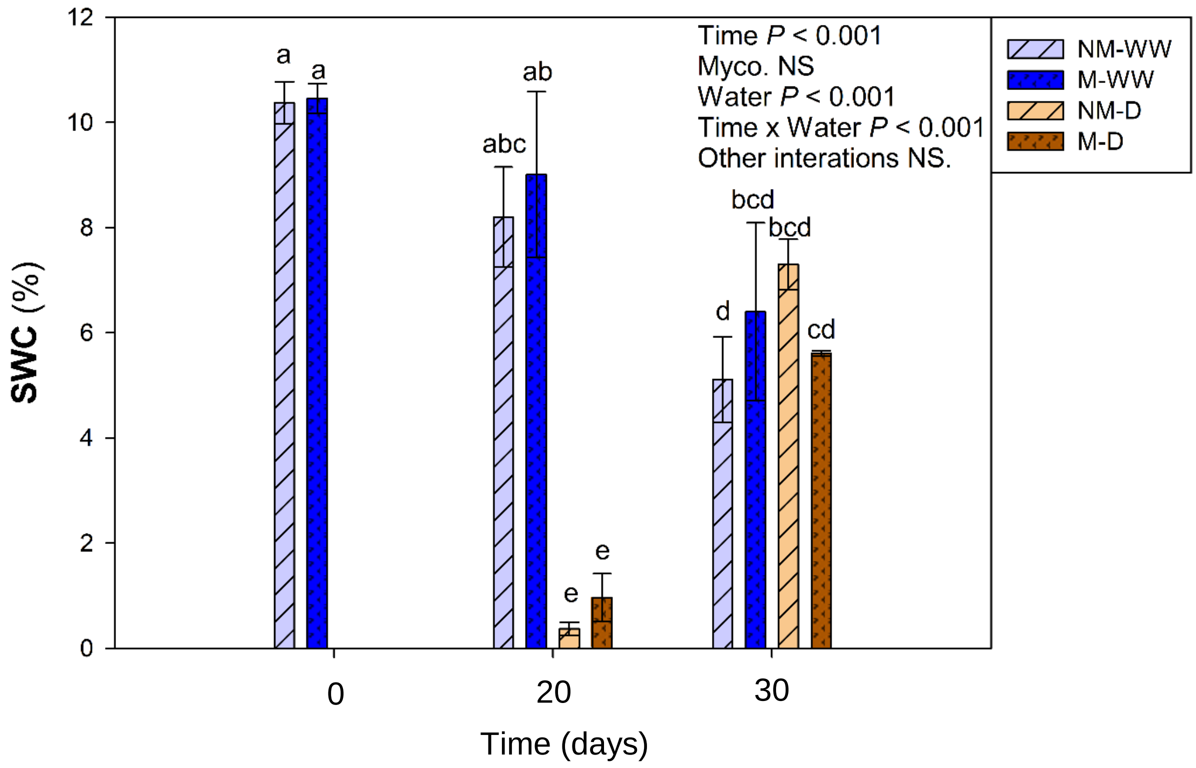

Supp. Figure 2. Soil water content (SWC) of pots containing mycorrhizal (M) and non-mycorrhizal (NM) *Trifolium repens* plants under well-watered (WW) and drought (D) conditions. Homogeneous sand samples were taken during a progressive water stress up to 20 days after the beginning of the treatment, followed by a recovery period of 6 days. Data are means ± SEM of n=3 individual pots. To evaluate treatment effects, a three-way ANOVA for independent samples was performed, followed by a post-hoc Duncan test with different letters indicating significant differences between groups ($p<0.05$).
